# Device-based percutaneous treatments to decompress the left atrium in heart failure with preserved ejection fraction

**DOI:** 10.1007/s10741-022-10280-4

**Published:** 2022-11-19

**Authors:** Mauro Riccardi, Daniela Tomasoni, Enrico Vizzardi, Marco Metra, Marianna Adamo

**Affiliations:** Department of Medical and Surgical Specialties, Radiological Sciences, ASST Spedali Civili Di Brescia, and Public Health University of Brescia, CardiologyBrescia, Italy

**Keywords:** Heart failure with preserved ejection fraction, HFpEF, Interatrial shunt device, V-Wave Shunt, Atrial flow regulator

## Abstract

Heart failure with preserved ejection fraction (HFpEF) accounts for more than half of heart failure hospital admissions in the last years and is burdened by high mortality and poor quality of life. Providing effective management for HFpEF patients is a major unmet clinical need. Increase in left atrial pressure is the key determinant of pulmonary congestion, with consequent dyspnoea and exercise limitation. Evidence on benefits of medical treatment in HFpEF patients is limited. Thus, alternative strategies, including devices able to reduce left atrial pressure, through an interatrial communication determining a left–right shunt, were developed. This review aims to summarize evidence regarding the use of percutaneous interatrial shunting devices. These devices are safe and effective in improving hemodynamic and clinical parameters, including pulmonary capillary wedge pressure, 6-min walking distance, and New York Heart Association functional class. Data on cardiovascular mortality and re-hospitalization for heart failure are still scarce.

## Introduction

The prevalence of heart failure (HF) is approximately 1–2% in adults, and the overall incidence of HF is increasing, most notably in the context of an ageing population [1–3]. HF with preserved ejection fraction (HFpEF) already accounts for more than half of all HF hospital admissions [4, 5]. In the outpatient setting, the European Society of Cardiology (ESC) Long-Term Registry reports that 60% have HF with reduced ejection fraction (HFrEF), 24% have HF with mildly reduced ejection fraction (HFmrEF), and 16% have HFpEF [1, 6]. Patients with HFpEF present functional limitation, poor quality of life (QoL), and a higher mortality compared to healthy age-matched population [7–10]. Several studies also reported similar outcomes in HF patients irrespective of left ventricular ejection fraction (LVEF), with an annual mortality rate ranging from 1.3 to 24% [11–13].

Medical treatment has demonstrated to improve cardiac function, symptoms, and prognosis in patients with HFrEF, but failed to demonstrate benefits in HFpEF in large-scale clinical trials [1, 14–23]. To date, only diuretics are recommended in patients with HFpEF and signs of congestion in order to alleviate symptoms of HF. Recently, empagliflozin was found to be effective in reducing the primary composite endpoint of cardiovascular (CV) death or hospitalization for HF in the Empagliflozin in Heart Failure with a Preserved Ejection Fraction (EMPEROR-Preserved) trial [3, 24]. Also, dapagliflozin improved patient-reported symptoms compared to placebo in the PRESERVED-HF trial and reduced by 18% the combined risk of worsening HF or CV death among patients with HF and an LVEF > 40% in the Dapagliflozin Evaluation to Improve the LIVEs of Patients With PReserved Ejection Fraction Heart Failure (DELIVER) trial [25–27].

However, the management and treatment of HFpEF still remains a major unmet clinical need [3]. Treatments alternative to medical therapies, including devices, may have a major role [28].

The aim of this review is to summarize evidence regarding the use of percutaneous interatrial shunting devices in patients with HFpEF.

## The role of left atrial pressure and the rationale for the development of interatrial shunt devices

**Fig. 1:**
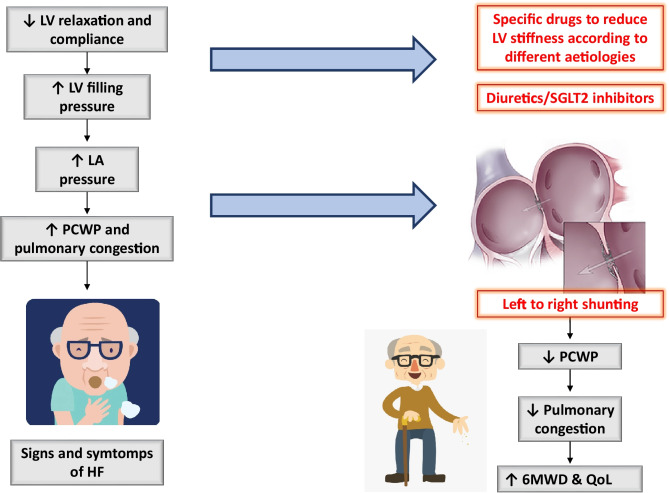
Pathophysiologic mechanisms leading to dyspnoea in patients with HFpEF and possible targets for therapies, including the interatrial shunt devices. 6MWD, 6-min walking distance; HFpEF, heart failure with preserved ejection fraction; LA, left atrium; LV, left ventricle; QoL, quality of life; PCWP, pulmonary capillary wedge pressure; SGLT2, sodium/glucose cotransporter 2

Impaired left ventricular (LV) relaxation and compliance are the hallmark features of HFpEF. They lead to elevated LV filling pressure and left atrial pressure (LAP) at rest or during exercise and may cause pulmonary hypertension (PH), with consequent exertional dyspnoea and exercise limitation or pulmonary oedema (Fig. 1) [29–34].

A rise in LAP results in atrial remodelling and failure. Left atrial (LA) disease has been recently introduced in the ESC guidelines for the management of HF and defined as a complex of subclinical structural, electrophysiological, and functional changes that affect the atria with the potential to produce clinical consequences [1]. LA disease causes HF symptoms, an increase risk of atrial fibrillation (AF), right ventricular (RV) dysfunction, impaired exercise capacity, and adverse outcomes [35]. Higher LAP, measured as pulmonary capillary wedge pressure (PCWP) at rest or during exercise, has been associated with higher mortality and morbidity [36], whereas decongestion therapies guided by a real-time indirect monitoring of LAP resulted in a reduction of HF hospitalizations [37–39].

Unloading the LA by shunting blood to the lower pressure reservoir of the right atrium (RA) and systemic veins may reduce pulmonary venous pressure and improve symptoms and outcomes in these patients (Fig. 1). The hypothesis of a potential benefit of an iatrogenic interatrial shunt in HF was based on the observation that in the setting of mitral stenosis, a condition also associated with elevated LAP and LA dysfunction, the coexistence of a congenital atrial septal defect (named Lutembacher syndrome) was associated with fewer symptoms and a more favourable clinical course, without exceeding the risk of right HF or stroke [40]. On the other hand, the closure of atrial septal defects in patients with unrecognized LV dysfunction may lead to abrupt elevation of LAP and pulmonary oedema [41].

A series of devices, creating a left-to-right controlled interatrial shunt, have been developed to decompress the LA in HF patients [32, 42–44]. The shunts work as on-demand, self-regulating LAP lowering systems, according to the pressure gradient between the LA and RA [45]. As LAP increases in response to any conditions (exercise, rise in systolic blood pressure, etc.), a small amount of LA blood is shunted to the RA, leading to a reduction in the LAP. The interatrial shunt creates a mismatch between pulmonary and systemic flow (Qp:Qs), possibly determining a risk of worsening right-sided HF. However, if the shunted volume is limited, the reduction in LAP overcomes the increase in right-sided volume, and pulmonary artery and right heart pressures remain unchanged or may be reduced as well. Traditionally, Qp:Qs ratios < 1.5 are known to be well-tolerated and without deleterious right-sided changes. Thus, the therapeutic interatrial shunt ratio (Qp:Qs) goal should be around 1.2 or slightly greater.

**Fig. 2:**
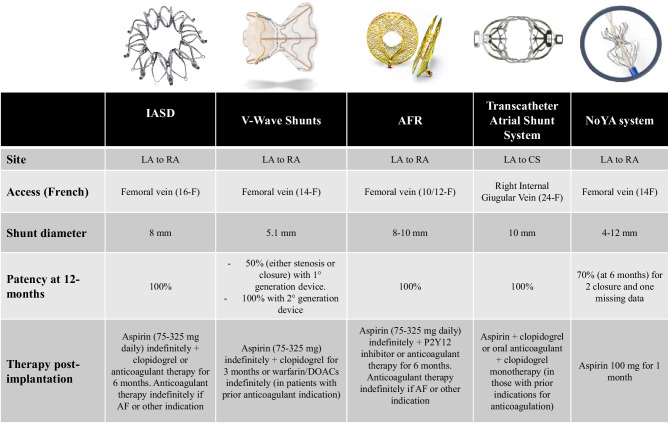
The main characteristics of devices used to reduce left atrial pressure in patients with HFpEF. AF, atrial fibrillation; AFR, atrial flow regulator; CS, coronary sinus; DOACs, direct oral anticoagulants; LA, left atrium; IASD, interatrial shunt device; RA, right atrium

The following sections will describe devices which are currently under investigation in patients with HFpEF. Some of them already received CE approval (Tables 1 and 2, Fig. 2).

**Table 1:** Overview of trials on the use of device-based percutaneous treatments to decompress the left atrium in heart failure with preserved ejection fraction

Author, publication’s date	Trial design	Patient with HFpEF (number (%))	Main inclusion criteria	Endpoints	Safety (procedural success rate)	Efficacy	Survival and HFH
Interatrial shunt device							
Søndergaard et al., European Journal of Heart Failure (2014) [49]	Pilot trial/phase I, prospective, single arm, unblinded, multicentre	*n* = 11 (100%)	LVEF > 45%, ≥ 1 HFH or NYHA class III/IV, baseline PCWP at rest ≥ 15 mmHg or PCWP exercise ≥ 25 mmHg	Primary endpoint: SADE at 30 days. Secondary endpoint: procedural success and clinical efficacy at 30 days	No SADE at 30 days (100%)	At 30 days, LV filling pressures were reduced by 5.5 mmHg (*p* = 0.005) with improvement in NYHA functional class, QoL, and 6MWD	No deaths during follow-up. One patient (0.09%) had HFH
REDUCE LAP-HF, Hasenfuss et al., Lancet 2016 [51]	Phase I, prospective, single arm, unblinded, multicentre	*n* = 68 (100%)	Symptomatic HFpEF (LVEF > 40%), PCWP at rest ≥ 15 mmHg or PCWP exercise ≥ 25 mmHg	Primary endpoint: safety and performance of the IASD at 6 months. Secondary endpoint: clinical efficacy at 6 months	No SADE at 6 months (97%)	Mean exercise PCWP was lower than at baseline both at 20-W workload and at peak exercise. Median NYHA functional class had improved from III to II (*p* < 0·0001); mean 6MWD from 313 to 345 m (*p* = 0·0023)	No deaths during follow-up; 9 patients (14%) had an HFH within 6 months
Kaye et al., Circulation, 2016 [52]	1-year follow-up of REDUCE LAP-HF trial	*n* = 64 (100%)	Symptomatic HFpEF (LVEF > 40%), PCWP at rest ≥ 15 mmHg or PCWP exercise ≥ 25 mmHg	Primary endpoint: safety and performance of the IASD at 1 year. Secondary endpoint: clinical efficacy at 1 year	No SADE at 12 months (100%)	Sustained significant reduction in workload indexed PCWP and significant improvements in NYHA functional class, QoL, and 6MWD	3 patients died (overall 1-year survival of 95%). 17 HFH occurring in 13 patients (20%)
REDUCE LAP-HF I, Feldman et al., Circulation. 2018 [32]	Phase 2, prospective, randomized, parallel-group, blinded sham-controlled	*n* = 44 (89%)	LVEF ≥ 40%, NYHA class III or ambulatory class IV, exercise PCWP ≥ 25 mmHg, and PCWP-RAP gradient ≥ 5 mmHg	Primary endpoints: change in PCWP during exercise, peri-procedural events and MACE/renal events at 1 month. Secondary endpoints: clinical efficacy and change in peak exercise PCWP at 1 month	No SADE at 1 month (95.5%)	At 1 month, the IASD resulted in a greater reduction in PCWP compared with sham control (*p* = 0.028). Peak PCWP decreased by 3.5 ± 6.4 mmHg in the treatment group versus 0.5 ± 5.0 mmHg in the control group (*p* = 0.14)	No deaths or HFH at 1 month in the treatment arm
REDUCE LAP-HF II, Shah SJ et al., Lancet 2022 [54]	Phase 3, prospective, randomized, blinded, sham-controlled	*n* = 626 (93%)	Symptomatic HF, LVEF ≥ 40%, PCWP exercise ≥ 25 mmHg while exceeding RAP ≥ 5 mmHg	Primary endpoint: hierarchical composite of CV death or non-fatal ischemic stroke at 12 months, rate of total HF events up to 24 months, and change in KCCQ overall summary score at 12 months. Secondary endpoints: rate of HFH, safety, and clinical benefits	No differences in the composite safety endpoint between the two groups. However, patients treated with the shunt were more likely to have a MACE: 4% vs. 1%, *p* = 0·025 (100%)	The primary efficacy endpoint did not differ between the groups, and there were no differences in the individual components of the primary endpoint. NYHA class improved to a greater extent in the shunt device–treated patients than sham-treated controls (*p* = 0.006)	CV death was uncommon (3 vs. 2, *p* = 0.65) and the total rate of HFH was similar between the two groups (0.28 events per patient-year vs. 0.25, *p* = 0.45)
V-Wave Shunt							
Rodés-Cabau et al., J Am Coll Cardiol Intv, 2018 [57]	Phase 1, prospective, single arm, unblinded, multicentre	*n* = 38 (21%)	HFrEF or HFpEF, NYHA functional class III/IV, HFH prior 12 months or NT-proBNP levels ≥ 1500 pg/mL	Primary endpoints: SADE at 3–12 months and procedural success. Secondary endpoints: clinical outcomes at 3–12 months	One cardiac tamponade successfully treated with pericardiocentesis (97%)	At 3 months, 78% of patients improved from NYHA functional and at 12 months, 60% continued to be improved (*p* < 0.001). For QoL, 74% and 73% of patients improved by ≥ 5 points at 3 and 12 months, respectively (*p* < 0.001). The 6MWD increased by 41 m at 3 months (*p* = 0.004) and by 28 m at 12 months (*p* = 0.048). Improvements in PCWP (from 23.3 ± 5.4 mmHg at baseline to 18.0 ± 4.0 mmHg at 12 months, *p* = 0.011) were found	Throughout the total follow-up duration, there were 10 deaths (8 CV and 2 of non-CV causes). One patient received a LVAD at 15 months, and another underwent HT at 27 months. There were 30 HFH events in 11 patients (29%), and 45 non-HFH in 19 patients (50%). Of the non-HFH, 14 (31%) had coexisting WHF
Guimaraes et al., Eurointervention 2020 [58]	First-in-human experience with 2° generation of V-Wave Shunt	*n* = 10 of which 6 complete the 1-year follow-up (16%)	HFrEF and HFpEF with a history of chronic HF and NYHA class ≥ III despite OMT	MACE and HFH	1 patient died in the hours following the procedure due to an electrical storm	83% improved NYHA class (*p* = 0.013), KCCQ score improved from 51.1 ± 9.7 at baseline to 83.0 ± 15.4 at 1 year (*p* = 0.006), and 6MWD improved from 274 ± 65 to 338 ± 104 (*p* = 0.169)	3 patients died during the 1-year follow-up (one for advanced HF at 10 months and two for pneumonia and 6.9 and 5.5 months, respectively). The first two patients mentioned above also had an HFH
Atrial flow regulator							
AFR-PRELIEVE, Paitazoglou et al., European Journal of Heart Failure, 2021 [63]	Pilot study, prospective, non-randomized, open-label, multicentre	*n* = 53 (54%)	HFrEF or HFpEF, NYHA functional class III/IV, PCWP at rest ≥ 15 mmHg or PCWP during exercise ≥ 25 mmHg	Primary endpoint: the rate of SADE at 3 months. Secondary endpoint: SADE at 12 months, clinical efficacy at 1 year	There was one device embolization into the LA, which required surgical removal. One patient experienced a SADE due to post-procedural bleeding and syncope (96%)	At 3 months, rest PCWP decreased by 5 (− 12, 0) mmHg (*p* = 0.0003). NYHA class and QoL improved significantly at 1 year in all patients (NYHA class decrease by 1, *p* < 0.0001; KCCQ overall summary score + 14.9 (0.6, 38), *p* < 0.0001). 6MWD improved significantly in the whole patient collective (6MWD at 1 year + 50 m, *p* = 0.0198)	Six (11%) patients had HFH, and three (6%) patients died
Transcatheter atrial shunt system							
Simard et al., JACC Cardiovascular Interventions, 2020 [65]	First-in-human experience	*n* = 11 (64%)	HF with NYHA functional classes III–IV, PCWP > 15 mmHg at rest with a gradient between PCWP and RAP > 5 mmHg	First data on safety and efficacy	No SADE at 1 month (procedural success rate 73%)	NYHA functional class improved to I or II in 87.5%, KCCQ score improved in 66.7%, while 6MWD improved significantly in 42.9% of patients. PCWP was reduced at 100 days (− 9 mmHg;) with diminished LA-to-RA gradients (− 10 mmHg)	2 deaths (20%) during follow-up (one at 221 days and the other at 450 days). No HFH (median follow-up 261 days)
Radiofrequency ablation–based interatrial shunt							
Sun et al., Circulation: Heart Failure (2022) [67]	First-in-man trial	*n* = 10 (90%)	HFpEF or HFmrEF with NYHA functional classes II–IV and at least one HFH during the last years though OMT, NT-proBNP > 200 pg/mL (or > 600 if AF) and LAP > 15 mmHg	Primary endpoints: SADE and interatrial shunt performance. Secondary endpoints: HFH and the change in clinical functions at 6 months	No SADE during the whole study (100%)	Shunt could still be observed in 7 patients at 6 months (2 patients showed the closure and 1 patient missed follow-up). NT-proBNP was reduced by 2149 pg/mL (*p* = 0.028), 6MWD was increased by 88 m (*p* = 0.008) and NYHA class was improved in 8 patients. mean decrease of 3.1 ± 3.9 mm in the LAD (*p* = 0.042)	No deaths or HFH at 6 months

*6MWD*, 6-min walking distance; *CV*, cardiovascular; *HF*, heart failure; *HFH*, heart failure hospitalization; *HFmrEF*, heart failure with mildly reduced ejection fraction; *HFpEF*, heart failure with preserved ejection fraction; *HFrEF*, heart failure with reduced ejection fraction; *HT*, heart transplantation; *IASD*, interatrial shunt device; *KCCQ*, Kansas City Cardiomyopathy Questionnaire; *LA*, left atrium; *LAD*, left atrial dimension; *LAP*, left atrial pressure; *LV*, left ventricle; *LVAD*, left ventricular assist device; *LVEF*, left ventricular ejection fraction; *MACE*, major adverse cardiovascular events; *NYHA*, New York Heart Association; *NT-proBNP*, N-terminal pro-hormone brain natriuretic peptide; *OMT*, optimal medical therapy; *PCWP*, pulmonary capillary wedge pressure; *QoL*, quality of life; *RA*, right atrium; *RAP*, right atrial pressure; *SADE*, serious adverse device events; *WHF*, worsening heart failure.

**Table 2:** Ongoing trials on the use of device-based percutaneous treatments to decompress the left atrium in heart failure with preserved ejection fraction

Author, publication’s date	Trial design	Estimated number of patients	Main inclusion criteria	Endpoints	Recruitment status
Interatrial shunt device					
REDUCE LAP-HF III [NCT03191656]	Post-market, prospective, observational	*n* = 500	Patients with HFpEF or HFmrEF with elevated LAP, who remain symptomatic despite standard OMT, in accordance with CE mark–approved labelling	Primary endpoints: MACE at 30 days; improvement in NYHA classification and in KCCQ score at 12 months	Recruiting
REDUCE LAP-HF IV [NCT04632160]	Prospective, multicentre, open-label, single arm	*n* = 150	Symptomatic HF, LVEF ≥ 40%	Incidence of and time-to-cardiovascular mortality or first non-fatal, ischemic stroke through 12 months; total HFH or visits for intravenous diuretics up to 24 months and time-to-first HF events; change in KCCQ at 12 months	Withdrawn pending formal analysis of REDUCE LAP-HF II Pivotal Study results
V-Wave Shunt					
RELIEVE-HF [NCT03499236]	Phase 3, multicentre, 1:1 randomized, blinded	*n* = 500	HFrEF or HFpEF, HFH prior 1 year or NT-proBNP ≥ 1500 pg/mL with NYHA classes III–IV or HFH prior 1 year and NT-proBNP > 1500 pg/mL with NYHA II	Primary endpoints: major device-related adverse events at 30 days; effectiveness-hierarchical composite of death, heart transplant or LVAD implantation, HFH, worsening HF events and change in KCCQ score Secondary endpoints: change in 6MWD and KCCQ score	Recruiting
Atrial flow regulator					
PROLONGER trial [43]	Phase 2, prospective, open-label, single-arm, unblinded	*n* = 30	HFrEF or HFpEF, NYHA classes III–IV, HFH prior 12 months, PCWP at rest > 15 mmHg or PCWP > 25 mmHg during handgrip test	Primary endpoint: clinical improvement at 12 months (at least 10% increase in 6MWD) Secondary endpoints: reduction in NYHA class at 12 months, device-related adverse events at 12 months, PCWP reduction at rest and during handgrip test 30 days after AFR implantation, KCCQ score within 12 months	Unknown
FROST-HF [NCT03751748]	Phase 2, multicentre, randomize, sham-controlled, single blind	*n* = 230	LVEF > 45%, NYHA class ≥ II or 6MWD < 80% predicted, LA enlargement, PCWP ≥ 25 mmHg during exercise, LAP ≥ 5 mmHg than RAP	Primary endpoint: 6MWD at 12 months Secondary endpoints: MACE, cardiac death, congestive HF, and KCCQ score at 12 months	Unknown
AFteR Registry [NCT04405583]	Multicentre, prospective, follow-up study	*n* = 100	Patients for whom AFR is indicated and planned	Primary endpoint: safety in the 1-year following implantation Secondary endpoints: device in situ after 3 years, evidence of left-to-right shunts at 3 years, change in QoL at 3 years	Recruiting
Transcatheter atrial shunt system					
ALt FLOW US [NCT03523416]	Phase 1, multicentre, prospective, early feasibility	*n* = 55	Chronic symptomatic HF, PCWP at rest > 15 mmHg or > 25 mmHg during exercise and LAP exceeds RAP by 5 mmHg at rest or 10 mmHg during exercise	Primary endpoints: MACE, or renal events; re-intervention for study device-related complications at 30 days Secondary endpoints: device and procedural success; improvement of PCWP and clinical success	Recruiting
Radiofrequency ablation and balloon dilation					
PAS trial [NCT04573166]	First-in-man study	*n* = 30	Symptomatic HF in NYHA class III or IV ambulatory, LVEF ≥ 45%, PCWP ≥ 18 mmHg at rest and PCWP mean RAP gradient ≥ 5 mmHg	Primary endpoints: distance in 6MWD and the size of created fenestration. Secondary endpoints: the percent of subjects who experience MACE; change of PCWP or LAP at rest; myocardial remodelling	Recruiting
Alleviant device					
Alleviate-HF-1 [NCT04583527]	First-in-man study	*n* = 15	LVEF ≥ 40%, NYHA functional classes II–IV, at least one HFH during the last year, echocardiographic evidence of diastolic dysfunction, elevated LAP with a gradient compared to RAP	MACE at 1 month	Completed
Alleviate-HF-2 [NCT04838353]	Phase 1, prospective, open-label, multicentre, single-arm, unblinded	*n* = 30	LVEF ≥ 40%, NYHA functional classes II–IV, at least one HFH during the last year, echocardiographic evidence of diastolic dysfunction, elevated LAP with a gradient compared to RAP	MACE and change in supine exercise PCWP at peak exercise (at 1 month and through 12 months)	Recruiting

*6MWD*, 6-min walking distance; *AFR*, atrial flow regulator; *CE*, European Community; *HF*, heart failure; *HFH*, heart failure hospitalization; *HFmrEF*, heart failure with mildly reduced ejection fraction; *HFpEF*, heart failure with preserved ejection fraction; *HFrEF*, heart failure with reduced ejection fraction; *KCCQ*, Kansas City Cardiomyopathy Questionnaire; *LA*, left atrium; *LAP*, left atrial pressure; *LVAD*, left ventricular assist device; *LVEF*, left ventricular ejection fraction; *MACE*, major adverse cardiovascular events; *NYHA*, New York Heart Association; *NT-proBNP*, N-terminal pro-hormone brain natriuretic peptide; *OMT*, optimal medical therapy; *PCWP*, pulmonary capillary wedge pressure; *QoL*, quality of life; *RAP*, right atrial pressure.

## Interatrial shunt device

Interatrial shunt device (IASD, Corvia Medical, Tewksbury, MA, USA) is a bare-metal nitinol frame device, creating a permanent 8-mm communication between the atria, allowing a physiological pressure-dependent left-to-right flow [46]. IASD is implanted using percutaneous trans-septal access via the femoral vein. The LA disc is exposed and retracted to the septum, and the RA disc is unsheathed to secure the device in place. The design of the device is based on predictive haemodynamic modelling which evaluated the relationship between shunt size and LAP reduction, based on human data. Particularly, the IASD targets a Qp:Qs of 1.3 [47].

IASD was firstly evaluated in a pilot study, assessing safety and efficacy in 11 patients with symptomatic HFpEF. The study showed that an IASD could be safely implanted. The legs of the device are flat on the LA side to minimize the risk of thrombus formation. After the procedure, patients were treated with aspirin, lifelong, and clopidogrel, generally for 6 months [48]. Patients with a history of AF were maintained on oral anticoagulants and clopidogrel at the discretion of the physicians. At 30-day follow-up, the devices remained patent, and LV filling pressures were reduced by 5.5 mmHg (19.7 ± 3.4 vs. 14.2 ± 2.7; *p* = 0.005) with evidence of early clinical benefit, improvement in New York Heart Association (NYHA) functional class, 6-min walking distance (6MWD), and QoL (Table 1) [49, 50].

These preliminary results were confirmed in the Reduce Elevated Left Atrial Pressure in Patients With Heart Failure (REDUCE LAP-HF) trial, a larger, non-randomized study, including 68 patients with LVEF > 40%. There were no peri-procedural complications, or major adverse cardiovascular events (MACE) or need for cardiac surgical intervention for device-related complications at 6-month follow-up. A significant reduction in exercise PCWP was observed at 6 months. Moreover, there was an improvement in functional and exercise capacity, whereas a modest increase in right heart cardiac output and RA pressure (RAP) was observed. Sustained device patency at 6 months was confirmed by left-to-right shunting (pulmonary/systemic flow ratio: 1.06 ± 0.32 at baseline vs. 1.27 ± 0.20 at 6 months, *p* = 0.0004) [51]. Follow-up of these patients was subsequently extended to 12 months, providing evidence of a sustained and meaningful clinical benefit, evaluated through QoL score, 6MWD, and NYHA functional class. Invasive hemodynamic studies performed in a subset of patients demonstrated a sustained reduction in the workload-corrected exercise PCWP (*p* < 0.01). Echocardiographic parameters at 12 months showed a modest but stable reduction in the LV end-diastolic volumes, without changes in LA and RA volumes. By contrast, a small but significant increase in the RV end-diastolic volumes raised some concerns [52].

The Transcatheter Interatrial Shunt Device for the Treatment of Heart Failure With Preserved Ejection Fraction (REDUCE LAP-HF I) trial was the first randomized, sham-controlled trial designed to determine the effectiveness of the IASD in patients with HFmrEF or HFpEF. The trial met its primary endpoint of effectiveness, with statistically significant lowering of PCWP during exercise at 1-month follow-up (*p* = 0.028). No major complications, including death, myocardial infarctions (MI), IASD occlusions or removals after the procedure, or strokes/transient ischemic attacks (TIA), were reported in either of the study arms. The rate of HF-related hospitalizations or emergency department/acute care facility visits requiring intravenous treatment was < 1% in the treatment arm compared with 9.1% in the control arm. However, the small sample size prevented to reach statistical significance [32].

The impact of IASD on CV mortality and HF events remained to be assessed. In patients treated with this device in the open-label REDUCE LAP-HF cohort, Kaye et al. observed a 33% lower mortality rate than that predicted by the Meta-analysis Global Group in Chronic Heart Failure (MAGGIC) prognostic model over the entire observation period [53].

The atrial shunt device for heart failure with preserved and mildly reduced ejection fraction (REDUCE-LAP II) was a prospective, randomized, multicentre, blinded, sham-controlled trial, enrolling 626 patients with symptomatic HF, a LVEF of at least 40%, PCWP during exercise ≥ 25 mmHg while exceeding RAP by at least 5 mmHg. Patients were randomly assigned (1:1) to receive either a shunt device or sham procedure. The primary endpoint was a hierarchical composite of CV death or non-fatal ischemic stroke at 12 months, rate of total HF events up to 24 months, and change in Kansas City Cardiomyopathy Questionnaire (KCCQ) overall summary score at 12 months. The trial failed to demonstrate difference between groups for the primary endpoint (win ratio 1.0 [95% CI 0.8–1.2]; *p* = 0.85) and in the individual components of the primary endpoint. There were no differences in the composite safety endpoint between the two groups (*n* = 116 [38%] for shunt device vs. *n* = 97 [31%] for sham procedure; *p* = 0.11), but MACEs were more common in the atrial shunt group compared with the sham control (*p* = 0.025). Patency was 100% at 12 months [54].

Ongoing studies investigating IASD (NCT03191656, NCT04632160) are reported in Table 2. Since the device has received CE approval in the Europe, the REDUCE LAP III (NCT03191656) will collect post-market data in consecutive patients with HF treated with the IASD System II, to further evaluate efficacy, safety, and QoL outcomes in a real-world setting.

## V-Wave Shunt

The V-Wave Shunt device (V-Wave Ltd., Caesarea, Israel) consists of an hourglass-shaped nitinol frame encapsulated with a partially expanded polytetrafluoroethylene cover serving as an anchor for 3 porcine pericardial leaflets held together using a Prolene suture [46, 55]. The device is delivered via femoral vein and interatrial septal puncture. In the first design, a one-way bioprosthetic valve ensured only left-to-right shunting. A first-in-man experience with this device demonstrated initial safety and early beneficial clinical and haemodynamic outcomes in patients with HFrEF [56]. In a single-arm open-label study, enrolling HFrEF and HFpEF patients with NYHA functional class III or IV, interatrial shunting with the V-Wave system was safe and feasible (Table 1). However, a high rate of shunt stenosis (36%) or occlusion (14%) at 12 months, likely due to early valve degeneration, resulted in loss of efficacy [57].

These observations prompted the creation of a second-generation device, which preserved the hourglass shape but eliminated the valve component; instead, a 5.1-mm central opening was created. This device was tested in a pilot study including 10 patients with chronic HF and NYHA class ≥ III, despite optimal tolerated drug and device therapies [58].

The ongoing RELIEVE-HF (Reducing Lung Congestion Symptoms using the V-Wave Shunt in Advanced Heart Failure) (NCT03499236) trial will evaluate safety and efficacy of the second-generation V-Wave Shunt in patients with advanced HF (NYHA functional class III or IV), regardless of LVEF. The co-primary endpoints are the frequency of major device-related adverse events (time frame: 30 days after randomization) and a hierarchical composite of death, heart transplant or left ventricular assist device (LVAD) implantation, HF events, and change KCCQ (time frame: follow-up duration at endpoint analysis ranges from a minimum of 12 to a maximum of 24 months) (Table 2). The trial aims to randomize 500 patients. First results on 97 patients showed high implantation success rates and safety, as well as sustained improvements in QoL from the first month. Moreover, shunt patency through 12 months was 100% [57].

## Atrial flow regulator

The atrial flow regulator (AFR, Occlutech, Istanbul, Turkey) is a self-expandable nitinol mesh braided into 2 flat discs creating a 1- to 2-mm fenestrated neck. As previous devices, it is delivered via femoral venous access following a trans-septal puncture. The central opening can have various diameters (6, 8, and 10 mm). However, only the 8-mm and 10-mm diameters obtained the CE mark for HF patients. The device is designed to allow interatrial bidirectional flow [42, 46, 59]. Due to the possibility of creating a bidirectional shunt, AFR was initially successfully tested in patients with severe, irreversible pulmonary arterial hypertension (PAH) [59, 60].

The Pilot Study to Assess Safety and Efficacy of a Novel Atrial Flow Regulator in Heart Failure Patients (PRELIEVE) was an open-label, prospective, non-randomized, first-in-man study investigating the feasibility up to 1-year follow-up of AFR implantation (8 mm or 10 mm diameters), in patients with HFrEF (*n* = 24) or HFpEF (*n* = 29) [61]. Among inclusion criteria, PCWP was ≥ 15 mmHg at rest or ≥ 25 mmHg during exercise. At 3 months, rest PCWP decreased by 5 mmHg (*p* = 0.0003) in the whole cohort. When analysed separately, the PCWP change was significant for HFpEF patients as compared with HFrEF patients. RAP remained unchanged after 3 months. Echocardiographic data at 12-month follow-up showed that LA/LV diameter and LVEF remained unchanged, with significant improvement of the *E*/*E*′ ratio, a parameter that reflects PCWP [62]. A mild significant dilatation of RV diameter was observed in the HFpEF cohort due to increased volume, although without deterioration of right heart function. The authors also observed an improvement of NYHA functional class, QoL, and 6MWD at 1 year. Shunt patency with unidirectional left–right shunting was proven in all patients with sufficient echocardiography readout at both 3 and 12 months [63]. After the procedure, aspirin (75–325 mg daily) indefinitely associated with a P2Y12 inhibitor or anticoagulant therapy (warfarin or a direct-acting oral anticoagulant) for 6 months has been recommended (empirically) [48].

Currently, several studies are ongoing to further test the safety and efficacy of the AFR (Table 2). The “Flow Regulation by Opening the Septum in Patients With Heart Failure Trial (FROST-HF)” trial (NCT03751748) will enrol 230 patients with LVEF > 45%. Furthermore, receiving CE approval, a large observational registry (Follow-up Study to Monitor the Efficacy and Safety of the Occlutech AFR in Heart Failure Patients [AFteR] Registry) (NCT04405583) will include patients undergoing AFR implantation for the monitoring up to 3 years after the procedure.

## Novel perspectives

Devices capable of creating interatrial shunts have proven to be feasible, safe, and effective. In a recent meta-analysis including 6 studies (5 single-arm open-label studies, 1 sham-controlled trial) and 226 patients with chronic HF, the pre-defined primary outcome of change in 6MWD from baseline to 12 months was improved by 28 m (95% confidence interval (CI) 10.9–45.3), without significant interaction between devices (*p* = 0.66) and LVEF subgroups (*p* = 0.21) [64].

All the aforementioned devices use the interatrial septum as the site of shunt placement. However, patients may develop a certain amount of right heart overload and enabling right-to-left shunting may lead to hypoxemia and systemic embolization. In addition, placement in the interatrial septum limits subsequent trans-septal punctures for percutaneous procedures [65].

In order to overcome these limitations, a novel approach has been proposed. The Transcatheter Atrial Shunt System (Edwards Lifesciences) was created to reduce PCWP, through the creation of a shunt from the LA to the coronary sinus (CS) (Fig. 2). It is a bare-nitinol implant with 4 arms and an internal shunting diameter of 7 mm. The device is deployed between the LA and the CS through a percutaneous atriotomy, a procedure involving CS cannulation from the right internal jugular vein, CS-to-LA puncture, and balloon dilation of the LA wall. Intraprocedural CS angiography, fluoroscopy, transoesophageal echocardiography, and hemodynamic assessment are performed to document appropriate device seating and LA-to-CS shunting. The shunt device is fully recapturable up until the point of final arm deployment, while deployed shunts can be closed using the commercially available Amplatzer Duct Occluder or Amplatzer Septal Occluder in the event of excessive shunting or evidence of RV compromise [65].

This device was initially tested in 11 patients with symptomatic HF (7 HFpEF, 4 HFrEF) despite maximally tolerated guideline-directed medical and device therapy and PWCP ≥ 15 mmHg with a gradient from PCWP to RAP > 5 mmHg. Among 8 patients undergoing successful implantation, a significant improvement in symptoms and haemodynamic parameters was observed [65, 66]. A prospective early feasibility clinical trial (ALt FLOW US) is ongoing to evaluate safety and efficacy of this device in a larger patient population (NCT03523416) (Table 2).

More recently, a radiofrequency ablation–based interatrial shunt (RAIAS) therapy with a novel non-implanted device was developed. This device, named the NoYA system (NoYA MedTech, Hangzhou, China), consisted of a self-expanded flowerlike nitinol stent fixed connected to the radiofrequency generator. The flowerlike stent was configured with a diameter adjustable from 4- to 12-mm waist in the middle, on which the electric poles were located. Under the radial force of the stent and the power of radiofrequency energy, an interatrial communication was made. After ablation, the device was removed from the body, leaving nothing but an artificial ASD [59]. This novel approach was evaluated first in 11 domestic pigs and then in 9 HFpEF patients and 1 with HFmrEF with NYHA functional classes II–IV [67]. RAIAS therapy was successfully administered to all patients, and an evidence interatrial left-to-right shunt flow was detected with a mean Qp:Qs of 1.3 ± 0.3. No major safety event was observed during the whole study. However, there was a progressive decreased in diameter of the defect from 5.0 mm post-procedure to 3.0 at 6 months, and two patients showed complete closure of the defect (1 of 3-month and 1 of 6-month follow-up), confirmed by transoesophageal echocardiography. Patients showed improvements of NYHA functional class (*p* = 0.003) at 6 months, a reduction of median NT-proBNP from 3533 to 1347 pg/mL (*p* = 0.028), and an improvement in 6MWD from 349 to 440 m (*p* = 0.008). In addition, echocardiographic parameters showed a mean decrease of 3.1 ± 3.9 mm in the LA diameter at 6 months compared with baseline (*p* = 0.042), reflecting the reduced overloading of the LA after RAIAS [67].

**Fig. 3:**
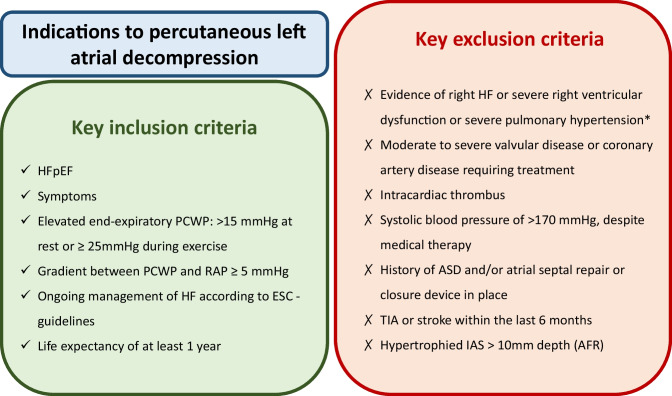
A practical guide for the selection of patients to be implanted with devices creating a left-to-right atrial shunt. Single asterisk indicates that right ventricular dysfunction is defined as TAPSE < 14 mm, RV volume ≥ LV volume, and PASP > 60 mmHg for AFR implantation; TAPSE < 12 mm or RVFAC ≤ 25% for V-Wave Shunt; and PVC > 20 mmHg or RAD > 45 mm, or TAPSE < 14 mm for radiofrequency ablation–based. ASD, atrial septal defect; AVR, aortic valve replacement; CABG, coronary artery bypass surgery; ESC, European Society of Cardiology; HFpEF, heart failure with preserved ejection fraction; LA, left atrium; MI, myocardial infarction; PCI, percutaneous coronary intervention; PCWP, pulmonary capillary wedge pressure; RAP, right atrial pressure; TIA, transient ischaemic attack

In conclusion, this first-in-man trial suggested that this new approach was a safe and feasible strategy for patients with HFpEF. Figure 3 summarizes the main criteria for the selection of patients that may benefit from interatrial shunt device implantation. However, future prospective randomized clinical trials are needed to clarify the efficacy and long-term safety.

Two further devices are being evaluated in HFpEF patients: one is able to create a personalized atrial septostomy through the combined use of radiofrequency ablation and balloon dilation (CURB) (NCT04573166); the other, the Alleviant System, a therapeutic interatrial shunt without a permanent heart implant (NCT04583527, NCT04838353).

## Conclusion

The management of HFpEF remains a challenge due to limited data about effectiveness of medical treatments. The use of devices capable of creating interatrial shunts, to reduce LAP, represents a promising therapeutic option. Early trials demonstrate feasibility, safety, and effectiveness in reducing PCWP, improving patients’ symptoms and QoL. Data on mortality and HF re-hospitalizations are still limited.

## Data Availability

Not applicable.

## Abbreviations

6MWD: 6-Min walking distance
AF: Atrial fibrillation
AFR: Atrial flow regulator
CS: Coronary sinus
CV: Cardiovascular
ESC: European Society of Cardiology
HF: Heart failure
HFmrEF: Heart failure with mildly reduced ejection fraction
HFpEF: Heart failure with preserved ejection fraction
HFrEF: Heart failure with reduced ejection fraction
IASD: Interatrial shunt device
KCCQ: Kansas City Cardiomyopathy Questionnaire
LA: Left atrium
LAP: Left atrial pressure
LV: Left ventricular
LVAD: Left ventricular assist device
LVEF: Left ventricular ejection fraction
MACE: Major adverse cardiovascular events
MAGGIC: Meta-analysis Global Group in Chronic Heart Failure
MI: Myocardial infarction
NYHA: New York Heart Association
PAH: Pulmonary arterial hypertension
PCWP: Pulmonary capillary wedge pressure
PH: Pulmonary hypertension
QoL: Quality of life
RA: Right atrium
RAIAS: Radiofrequency ablation–based interatrial shunt
RV: Right ventricular
TIA: Transient ischaemic attack
